# *Drosophila* Cellular Immunity Against Parasitoid Wasps: A Complex and Time-Dependent Process

**DOI:** 10.3389/fphys.2019.00603

**Published:** 2019-05-15

**Authors:** Chami Kim-Jo, Jean-Luc Gatti, Marylène Poirié

**Affiliations:** INRA, CNRS, Institut Sophia Agrobiotech, Université Côte d’Azur, Sophia Antipolis, France

**Keywords:** immunity, encapsulation, hematopoiesis, *Drosophila*, parasitoid wasp, venom, *Leptopilina*

## Abstract

Host-parasitoid interactions are among the most studied interactions between invertebrates because of their fundamental interest – the evolution of original traits in parasitoids – and applied, parasitoids being widely used in biological control. Immunity, and in particular cellular immunity, is central in these interactions, the host encapsulation response being specific for large foreign bodies such as parasitoid eggs. Although already well studied in this species, recent data on *Drosophila melanogaster* have unquestionably improved knowledge of invertebrate cellular immunity. At the same time, the venomics of parasitoids has expanded, notably those of *Drosophila*. Here, we summarize and discuss these advances, with a focus on an emerging “time-dependent” view of interactions outcome at the intra- and interspecific level. We also present issues still in debate and prospects for study. Data on the *Drosophila*-parasitoid model paves the way to new concepts in insect immunity as well as parasitoid wasp strategies to overcome it.

## Introduction

Eighty percent of known animal species are insects (Larsen et al., 2017), of which at least 20% have a parasitoid lifestyle (Forbes et al., 2018). The reproductive strategy of parasitoid species is unique among insects: they lay in or on other insects, their larvae developing by consuming host tissues, usually resulting in death. Most endoparasitoids are koinobionts, that is, their host can pursue its development with which their own offspring development will synchronize. They have thus evolved different strategies to circumvent the host immune response that, when successful, leads to the encapsulation of the parasitoid egg, i.e., its embedding by specialized hemocytes, together with melanization and production of Reactive Species (Carton et al., 2008; Nappi, 2010; Lu et al., 2014; Nakhleh et al., 2017). Encapsulation has notably been described in *Drosophila* species whose larvae are often attacked by endoparasitoid wasps (e.g. from the *Leptopilina*, *Ganaspis*, and *Asobara* genera) (Carton et al., 1986; Novković et al., 2011). Here, we will mainly rely on the *Drosophila melanogaster* interaction with *Leptopilina* wasps (Hymenoptera, Figitidae), one of the most advanced models for characterizing interaction mechanisms and the genetic bases of resistance and virulence (Dubuffet et al., 2009). It has helped to improve knowledge of hematopoiesis and cellular immunity in *Drosophila* and indirectly in other invertebrates. For thorough reviews of recent advances in *Drosophila* hematopoiesis, see Letourneau et al. (2016) and Banerjee et al. (2019). For a detailed follow-up of the cellular encapsulation of the wasp egg, see also Anderl et al. (2016).

## The *Drosophila* Encapsulation Response to Parasitism

**Figure 1:**
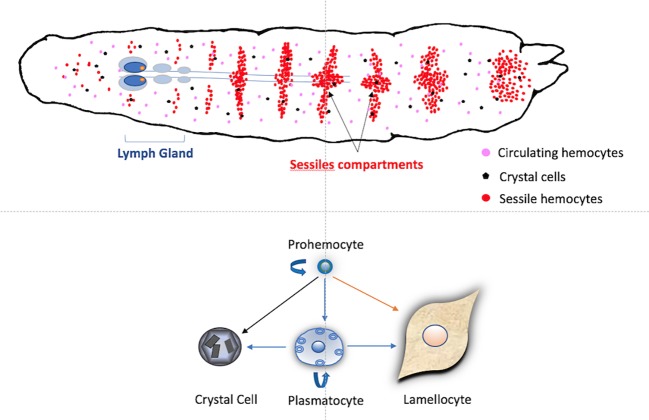
Schematic hematopoiesis in a *Drosophila melanogaster* larva. Hemocytes, mainly plasmatocytes and crystal cells, are circulating in the larval hemolymph. These circulating hemocytes can derive from embryonic prohemocytes or are also formed within the sessile compartment during most of the larval stages. This compartment is composed of sub-cuticular groups of cells, mainly prohemocytes and plasmatocytes, present in the different larval segments. At the end of the L3 stage, the first lobes of the lymph gland increase in size due to the proliferation of hemocytes that will be released just after pupation in healthy larvae. In normal conditions, self-renewing prohemocytes are considered as progenitors for the three main hemocytes types. After parasitoid oviposition, circulating or sessile plasmatocytes can also proliferate and transdifferentiate into crystal cells and lamellocytes. In parasitized hosts, lamellocytes can also differentiate from prohemocytes within the lymph gland and be released earlier than pupation to participate in the encapsulation.

**Figure 2:**
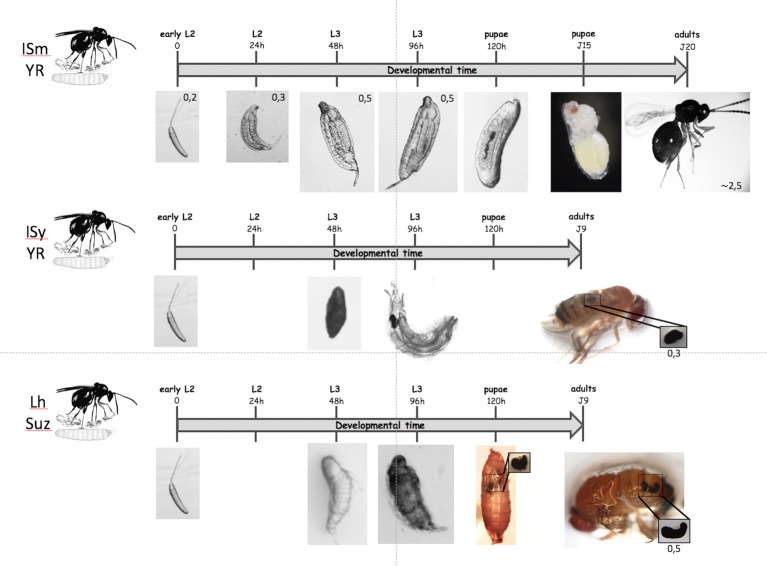
Different possible outcomes for parasitoids. On the top: when a *D. melanogaster* L2 larva is parasitized by the virulent *L. boulardi* ISm line, the parasitoid egg develops normally and the parasitoid larva hatches from its chorion 24 to 48 h later after parasitism. The parasitoid larva continues its moderate growth until the fly larva pupates and after a few days the puparium is mainly occupied by the parasitoid that has eaten almost all the fly larva tissues. Twenty days after oviposition, an adult parasitoid egress from the pupal case instead of a fly. On the center: when a *D. melanogaster* resistant L2 larva is parasitized by the avirulent *L. boulardi* G486 line, the parasitoid egg is rapidly encapsulated and a melanized capsule is formed after 48 h that remains visible in the larva. The capsule is retrieved in the emerging adult fly. On the bottom: when a *D. suzukii* larva is parasitized by the generalist *L. heterotoma* wasp, the egg hatches and the parasitoid larva remains alive after 48 h, with no observed capsule or melanisation. After 96 h, the parasitoid larva is dead or dying and is slowly embedded in a cell-formed melanized capsule. The capsule continues to enlarge until pupation and it is well visible through the pupal case and then retrieved in the abdomen of the emerging fly. The upper scale indicates the development time from oviposition and the observed stages. The scales provided on the pictures are given in millimeters (mm).

In *D. melanogaster*, the first noticeable events after wasp oviposition are the increased number of circulating plasmatocytes followed by the appearance of circulating lamellocytes – a hemocyte type produced in response to parasitism (Figure 1) – and activation of the phenoloxidase (PO) cascade (Carton et al., 2008, 2009; Nappi, 2010). Plasmatocytes form a first layer of cells surrounding the parasitoid egg to which the lamellocytes adhere, forming tight junctions, giving rise to a multilayer melanized capsule that is completed 48 h post parasitism (Figure 2; Russo et al., 1996; Bajgar et al., 2015). The production of cytotoxic radicals *via* the PO cascade is supposed to kill the parasitoid egg (Nappi and Vass, 1993; Nappi et al., 1995). For this response to be effective, timing may be essential. The encapsulation should be completed in less than 48 h because at that time the parasitoid egg has hatched (see Figure 2), and a moving larva is more likely to escape from the forming capsule.

## The Controversial Origin of the Main Circulating Immune Cells that Form the Capsule

The number of circulating hemocytes increases during the *D. melanogaster* larval stages from a few hundred to around 7,000 (Lanot et al., 2001; Petraki et al., 2015). They come from embryonic progenitor cells, the prohemocytes, which circulate in the hemolymph or are found in the larval lymph gland or in subcuticular hematopoietic hubs called sessile compartment (Figure 1; Honti et al., 2010; Makki et al., 2010; Fauvarque and Williams, 2011; Gold and Brückner, 2015; Leitão and Sucena, 2015; Ghosh et al., 2018).

The three main types of differentiated hemocytes in *Drosophila* larvae are now considered as distinct lineages that are formed from the prohemocyte progenitor (Figure 1). 95% of the circulating cells are phagocytic plasmatocytes that clear pathogens and dead cells, participate in wound healing, and synthesize extracellular matrix proteins (Martinek et al., 2008) and antimicrobial peptides (Charroux and Royet, 2009). Larval plasmatocyte populations express different subsets of markers (Jung et al., 2005; Honti et al., 2014) and two different adults populations have distinct immune functions (Clark et al., 2011). Crystal cells – about 5% of circulating cells – are characterized by the expression of the specific marker Lozenge and harbor large paracrystalline inclusions which contain prophenol-oxidases (PPO1 and PPO2). Upon injury, activation of the JNK pathway and TNF homolog Eiger lead to their disruption and the release of PPO zymogens in the hemolymph (Bidla et al., 2007; Dudzic et al., 2015). The third cell type, the lamellocytes, are large flat cells with adherent properties that are rare in healthy larvae but can represent up to 50% of circulating hemocytes after wasp parasitism. They form the successive outer layers of the capsule and produce a specific pro-phenoloxidase (PPO3), required with PPO2 for a complete melanisation of the capsule (Irving et al., 2001; Dudzic et al., 2015). Interestingly, the PPO3 gene seems specific to the melanogaster subgroup, suggesting that PPO production by lamellocytes is a recently evolved defense mechanism against parasitic wasps (Salazar-Jaramillo et al., 2014; Dudzic et al., 2015). The precise tissue origin of lamellocytes involved in encapsulation is still debated: they can be derived from sessile or circulating plasmatocytes (Anderl et al., 2016) or released as mature lamellocytes following accelerated proliferation and bursting of the lymph gland (Letourneau et al., 2016). All these recent advances about hematopoiesis have benefited from the genetic tools in *Drosophila* and also from the identification of hemocytes surface markers such as P1 for plasmatocytes or L1/Atilla for lamellocytes (Kurucz et al., 2007). The use of markers has improved discrimination and the tracking of different cell types but also, coupled with cell cytometry, demonstrated the presence of transient precursor populations (Anderl et al., 2016).

### Lamellocyte Production From the Sessile Compartment

Until recently, *Drosophila* hemocytes were thought to be produced in two waves, one during embryogenesis and the other in the lymph gland at the end of the larval stage (Gold and Brückner, 2015). However, new studies suggest a much higher plasticity, with functional hematopoietic sites distributed along the larva and also present in the adult fly (Gold and Brückner, 2015; Ghosh et al., 2018). The larva thus contains two hematopoietic compartments, the sessile compartment and the lymph gland. Hemocytes in the sessile compartments proliferate (self-renewal) and differentiate into plasmatocytes that can transdifferentiate into crystal cells by a Notch signaling-dependent process (Márkus et al., 2009; Leitão and Sucena, 2015). These new hemocytes can reach the hemolymph to contribute gradually to the pool of circulating larval hemocytes. In healthy larvae, they are mainly released at the onset of metamorphosis but simple stimuli such as light brush strokes can induce this release, indicating that these hemocytes fate also depends upon systemic and/or local signals including neuronal ones (Makhijani and Brückner, 2012; Tokusumi et al., 2017). An immune challenge such as oviposition of wasp can also cause their premature mobilization and induce their trans-differentiation into lamellocytes (Márkus et al., 2009; Honti et al., 2010; Anderl et al., 2016). Indeed, few hours after parasitism, a novel population of cells derived from plasmatocytes, the lamelloblasts, appears in the circulation, proliferate actively and develop into lamellocytes (Anderl et al., 2016). Plasmatocytes transdifferentiation into lamellocyte-like cells can also occur directly on the wasp egg. Different signaling pathways are important for the regulation of lamellocytes proliferation upon parasitism (Letourneau et al., 2016), including the Toll and Jak-Stat pathways (Morin-Poulard et al., 2013; Louradour et al., 2017). Activation of Jak-Stat induces the Charlatan (*Chl*) protein (a transcription factor that interacts with CoREST, a transcription repressor complex exhibiting histone deacetylase and demethylase activities) that is involved in lamellocytes transdifferentiation from plasmatocytes (Stofanko et al., 2010).

### Lamellocyte Production From the Lymph Gland

At the end of the third stage, the lymph gland is composed of a large pair of primary anterior lobes organized into three domains: the cortical zone (CZ), the medullary zone (MZ), and the posterior signaling center (PSC), followed by pairs of small posterior lobes, each separated by a pair of pericardial cells (Figure 1). The differentiation of lymph gland hemocytes starts during the third instar: First, the cortical zone (CZ) of the primary lobes expands due to prohemocytes proliferation and differentiation into plasmatocytes, a small number of crystal cells and occasionally a few lamellocytes. Then, the progenitors of the medullary zone (MZ) become quiescent. As development progresses, almost all lymph gland hemocytes differentiate, and 8 h after pupation, all cells have been released into the circulation (Grigorian et al., 2011). The proliferation and differentiation of hemocytes is controlled by a wide range of signals from the posterior signaling center (PSC), CZ, and MZ, and from systemic sources such as neurotransmitters and growth factors from the brain, and levels of nutritional compounds. In the PSC, high levels of reactive oxygen species (ROS) (Owusu-Ansah and Banerjee, 2009; Letourneau et al., 2016; Louradour et al., 2017), activation of the Wingless signaling pathway (Sinenko et al., 2009) and expression of the EBF transcription factor Collier (Benmimoun et al., 2012; Oyallon et al., 2016) are required for the maintenance of a pool of pluripotent progenitors. The PSC secretes also diffusible signals such as hedgehog (Hh) and the platelet-derived growth factor/vascular endothelial growth factor-like factor (PVF1) to activate different pathways in the lymph gland compartments (Mondal et al., 2011, 2014). Hh acts directly on the MZ progenitors to maintain them in their pluripotent state. PVF1 acts on differentiating hemocytes, stimulating the secretion of adenosine deaminase-related growth factor-A (ADGF-A), which leads to the inactivation of the adenosine/AdoR (adenosine receptor) pathway in MZ cells by modulating the extracellular adenosine level. This double control allows the maintenance of the balance between progenitors and differentiated cells. Other signaling pathways are involved such as JAK/STAT and Toll, or the insulin/IGF (IlS) and target of rapamycin (TOR) pathways and components of the nutrient detection system (Morin-Poulard et al., 2013; Shim et al., 2013). The prohemocyte fate is also controlled by local signals from the neighboring heart tube and through the regulation of the PSC morphology (Morin-Poulard et al., 2016).

Six hours after parasitic wasp infestation, hemocyte proliferation starts in the lymph gland leading to a massive differentiation of lamellocytes (Lanot et al., 2001). The burst of the lymph gland, releasing all hemocytes into the hemolymph, occurs after 24−48 h, depending on the experimental conditions and possibly the *D. melanogaster* strain used. The mechanism(s) responsible for the anticipated differentiation in the gland have not yet been fully identified, but they could be triggered by the production of ROS and the local immune response at the oviposition site (Sinenko et al., 2011). In response, the PSC secretes the epidermal growth factor receptor (EGFR) ligand Spitz that binds to receptor and activates the Ras/Erk pathway resulting in the appearance of dpERK-positive lamellocytes in circulation (Lusk et al., 2017). Expression of a dominant-negative form of EGFR in the lymph gland and circulating hemocytes suppresses the lamellocyte induction by wasp oviposition (Sinenko et al., 2011), suggesting a role of this pathway in lamellocyte differentiation also for the other compartments. Spitz is the major EGFR ligand, but other activators and inhibitors may participate whose roles have to be established in case of parasitism (Lusk et al., 2017). More and more evidence suggests that signals secreted by different tissues upon wasp oviposition play a role in encapsulation. For example, the release of the cytokine Edin from the fat body (Vanha-aho et al., 2015) or the activation of JAK/STAT signaling in somatic muscles induced by Upd2 and Upd3 secretion from hemocytes (Yang et al., 2015), both required for the larvae to mount a normal encapsulation response.

The origin of the lamellocytes forming the capsule is still debated. Indeed, although both sessile and lymph gland lamellocytes may participate, which participate the most and the most precociously in the formation of the capsule remains unanswered. Interesting elements have been brought on both sides without the issue really being decided today. Further development of specific labeling of the lamellocytes from the respective compartments (lineage sorting) should help to solve this issue.

## In Search for New Genes and Signaling Pathways Involved in Encapsulation

Most studies have used wide genomic analyses or mutant flies to decipher the pathways involved in hematopoiesis and encapsulation. The identification of new pathways could, however, also benefit from the analysis of resistance genes identified in natural populations. We work on *D. melanogaster* strains, one resistant (YR) the other susceptible (YS) to *Leptopilina boulardi* (strain G486; Carton et al., 1992) that differ mainly by their allele at a major “resistance” gene, *Rlb*, the resistant allele being dominant. *Rlb* was localized in a small region of chromosome 2R (Hita et al., 1999, 2006) that contains two genes: CG33136 and *edl*/*mae* (ETS-domain lacking)/(modulator of the activity of ETS). Interestingly, Edl interacts *via* its SAM (sterile alpha motif) domain with several ETS transcription factors (Baker et al., 2001) such as the transcriptional activator Pointed (mainly Pointed P2) and the repressor Yan/Aop (Vivekanand, 2018), both involved in the EGF pathway. When activated, the EGF signal triggers the mitogen-activated protein kinase/extracellular signal-regulated kinase (ERK) kinase (MEK) known as Rolled. Among many targets, rolled phosphorylates Pointed and Yan, thus modulating their function by changing their SAM domain binding affinity (Vivekanand, 2018). The universal output of the EGF pathway is induction of gene expression, mostly through the Pointed transcriptional activator. It regulates the proliferation and differentiation of many cell types in *Drosophila,* and Yan and Pointed have been involved in plasmatocyte and lamellocyte differentiation (Zettervall et al., 2004; Dragojlovic-Munther and Martinez-Agosto, 2013). It is thus possible, although still to be proven, that the balance between Edl, Yan and Pointed finely regulate hemocyte differentiation in case of parasitism, thus participating in *Drosophila* immune resistance.

## Variation in *Drosophila* Immune Response and Parasitism Outcome, A Question of Timing?

### Intraspecific Variation

The timing of increase of the hemocyte population in circulation is likely important for encapsulation success. When the total number of circulating hemocytes of the *D. melanogaster* YR and YS strains (see above) was compared post-parasitism by *L. boulardi* G486, an increase in hemocyte load occurred after 6 h for YR compared to 15 h for YS (Russo et al., 2001). Therefore, the earlier increase in hemocytes load in the YR strain might explain its resistance phenotype. Whether it is due to a differential regulation of the hematopoietic tissues response between resistant and susceptible strains will deserve further studies.

### Interspecific Variation

The response to parasitism of different *Drosophila* species may differ from that of *D. melanogaster*. Indeed, species of the *melanogaster* subgroup all produce lamellocytes that actively participate in the encapsulation, but very different cells are involved in other species such as the multinucleated giant hemocytes found in the *ananassae* subgroup (Márkus et al., 2015) or the nematocyte cell types in species from the *Drosophila* subgroup (Kacsoh et al., 2014; Bozler et al., 2017). The inducible production by parasitism of cells of different nature and origin in *Drosophila* species is intriguing from both a physiological and evolutionary point of view. We can indeed wonder about the mechanisms that led to this specific cellular evolution. Other *Drosophila* species from the *melanica* subgroup, while having lamellocytes, succeed in killing *L. heterotoma* eggs or larvae without forming a capsule, possibly through the production of cytotoxic radicals (Nappi and Streams, 1969; Carton et al., 2009). Another example of parasitoid elimination after the egg stage without encapsulation was provided by Paredes et al. (2016): it involves a lipid competition-based protection against some parasitoid wasp species conferred to the fly by the endosymbiont *Spiroplasma poulsonii*.

The immune response may also differ depending on the species of *Drosophila* even with the same cells involved. Indeed, the *D. suzukii* encapsulation response to *Leptopilina* wasp eggs shows a timing very far from that known in *D. melanogaster* (Iacovone et al., 2018). The immune response was not noticeable on *L. heterotoma* parasitoid eggs during the first 48 h (Figure 2) and only a thin coat of lightly colored cells wrapped the hatched larvae 72 h post-oviposition. Most parasitoid larvae died 96 h post-oviposition and were melanized, but large complete capsules were only observed at pupation and retrieved in emerged adult flies. Thus, although many hemocytes circulate in *D. suzukii* (Kacsoh and Schlenke, 2012) the immune response of this species seems largely delayed compared to that of *D. melanogaster*.

## How Parasitic Wasps Target the *Drosophila* Immune System

The advanced knowledge on *Drosophila* immunity is a great asset to decipher mechanisms ensuring parasitic success which, for *Drosophila* endoparasitoids, mainly relies on injection of venom containing immunosuppressive proteins (Poirié et al., 2014; Moreau and Asgari, 2015). The venom effect on the hemocytes, the lymph gland or the melanisation has been described for *Leptopilina* species (Dubuffet et al., 2009; Heavner et al., 2014). The *Leptopilina* and *Ganaspis* venom contains not only soluble proteins but also peculiar vesicles with an unclear biogenesis (Rizki and Rizki, 1990; Dubuffet et al., 2009; Ferrarese et al., 2009; Gatti et al., 2012), which are likely involved in parasitic success. These purified vesicles seemingly target the host lamellocytes, changing their shape from discoidal to bipolar, which supposedly prevent them to adhere and form a capsule, or inducing their lysis (Rizki and Rizki, 1984, 1990, 1994). Venomics analyzes allowed identifying the main venom proteins in *L. heterotoma* and *L. boulardi*, among which P40 associated with the vesicles of *L. heterotoma* (Heavner et al., 2017) and LbGAP (Colinet et al., 2013; Goecks et al., 2013), a Rho GTPase activating protein secreted in high amounts in *L. boulardi* and associated with the vesicles in the venom reservoir (Labrosse, 2003; Labrosse et al., 2005). LbGAP interacts *in vitro* with Rac1 and Rac2 (Colinet et al., 2007), both required for successful encapsulation (Williams et al., 2005, 2006), and it also immunolocalizes in lamellocytes of parasitized hosts (Colinet et al., 2007) like the *L. heterotoma* P40 protein (Chiu et al., 2006). The venom vesicles may therefore act as shuttles transporting virulence proteins and targeting them into *Drosophila* immune cells. Their mode of entry may represent a mechanism that shapes the host range of parasitoids, making it essential to decipher [e.g., identification of a specific receptor(s) on the lamellocytes] for understanding the host-parasitoid interaction in these species.

## Conclusion and Perspectives

Despite the recent rapid knowledge increases in insect cell immunity and encapsulation, particularly in the *Drosophila* model, some main gaps remain to be filled. The mechanisms of early recognition of a parasitoid egg as well as triggering and synchronization of the main actors of capsule formation are among the most important, as is the more precise identification of the role of ROS and NOS, factors from non-immune tissues. One can also mention the detailed formation of the capsule that will benefit from the development of markers to identify both the cell origin and type. On the parasitoids side, the ways of targeting the host immune compartments are diverse and the identification of molecular mechanisms remains a major challenge. Recent data have revealed a greater complexity of insect immunity against parasitoids than expected, with the presence of hematopoietic niches and the identification of pathways being reminiscent of vertebrate models (Sheehan et al., 2018). Finally, the natural variability of the host immune response and the mechanisms of virulence of parasitoids may open the way to new concepts. Understanding the short-term evolution of the immune interactions could well be announced as a major theme in the near future.

## Author Contributions

CK-J, J-LG and MP discussed the contents of the manuscript and contributed to the writing and editing.

### Conflict of Interest Statement

The authors declare that the research was conducted in the absence of any commercial or financial relationships that could be construed as a potential conflict of interest.
